# Research on controlling gas overrun in a working face based on gob-side entry retaining by utilizing ventilation type “Y”

**DOI:** 10.1038/s41598-023-36464-y

**Published:** 2023-06-06

**Authors:** Xihua Zhou, Zehao Jing, Yanchang Li

**Affiliations:** 1 College of Safety Science and Engineering, Liaoning Technical University, Huludao, 125000 Liaoning China; 2 Key Laboratory of Mine Thermodynamic Disasters and Control of Ministy of Education, Liaoning Technical University, Huludao, 125000 China

**Keywords:** Coal, Natural gas

## Abstract

To determine the characteristics of air leakage concerning a “Y” type ventilation in gob-side entry retaining with roof cutting, pressure relief, and the law of a resulted gas accumulation (GA), research is conducted by employing the CFD simulation incorporated with the gauged parameters of working face (WF) mining to analyze the air leakage of “Y” type ventilation. For this purpose, the 1201 fully mechanized coal mining face in the south Wu mining location of the Daxing coal mine is taken as an illustrative example to study the air leakage in the “Y” type ventilation. So, the gas concentration (GC) issue surpassing the limit in the upper corner of the goaf was simulated. The results show that the goaf is formed into an open space when roof cutting and pressure relief technology along the goaf is implemented. The air pressure at the upper corner of the WF would be the lowest, which is only 1.12 Pa. The airflow of air leakage under a pressure difference would move from the gob-side entry retaining to the goaf. Moreover, the simulation of mine ventilation indicates that the volume of air leakage positively correlates with the length of gob-side entry retaining. When the WF is advanced 500 m ahead, the maximum volume of air leakage would reach 247 m^3^/min within the range of 500–1300 m, and then the rate of air leakage gradually would decrease. When the WF is advanced at 1300 m, the air leakage would become the smallest, which is 175 m^3^/min. When gas control is under consideration, the effect of gas extraction would be best with the buried pipe whose depth and diameter are set to 4.0 m and 400 mm, respectively. So, the GC in the upper corner would become 0.37%. After the high-level borehole with a 120 mm diameter is mined, the GC in the deep goaf decreased to 3.52%, and the GC at the upper corner became further reduced to 0.21%. While the high-level borehole gas is extracted by employing the extraction system of the high-concentration gas, the extraction system of low-concentration gas is utilized to extract the upper corner gas of the WF, thus, the problem of gas overrun was resolved satisfactorily. During the recovery period of the mining, the GC at each gauging point was less than 0.8%, which effectively guided the secure production in the Daxing coal mine and provided a theoretical foundation to control gas overrun during the mining process.

## Introduction

Long-wall WFs are broadly employed in China’s coal mines, and their ventilation modes can be divided into types such as “U”, “Y”, “W”, etc. According to actual situations in mines and the characteristics of production in mines, a reasonable selection and scientific formulation of a ventilation mode and a ventilation plan could achieve a scheme for safer production^1–5^. A great deal of research has been done concerning those issues. Guo^6^ studied the impacts of “U” and “W” kind ventilations on the WF, then the goaf systematically was analyzed based on the data of GC and air volumes, for example, carbon dioxide in the WF. The “Y” type could better resolve the hidden danger of gas overrun in the upper corner of the WF and improve the working environment of a mine when compared with the “U” type^7–9^. Zhang^10^ used software to run numerical simulations to analyze the field of the gas flow in the goaf and proposed an extraction process utilizing a large-diameter borehole when partial “Y” type ventilation conditions exist. Jianhong^11^ calculated the volume of air leakage in the goaf and executed an investigation. The “W” type ventilation could decrease the pressure difference between the inlet and return air lanes in the goaf, thereby, air leakage was reduced. Gou^12^ proposed a mathematical model controlling the law of gas migration and the reduction of GC in the upper corner was effectively realized when the “Y” type was in use. Wang et al^13^ obtained not only the whole structure of the pores but also connected the coal parameters of the pore structure by employing 3D reconstructed CT images. It is suggested that the solid structure of coal is not fractal, and the porosity of coal samples decreases as connectivity raises. Tutak^14^ researched the problem of mine fires caused by air leakage in the “Y” type and put forward a judgment basis on air velocity and oxygen volume in the area in which the fire occurred, which proved that the coal having an oxidation degree in the goaf with a “U” type was found to be slightly larger than that of the “W” type. Zhu et al^15^ established the corresponding FLUENT model and studied the GC and the amount of gas extraction in the upper corner by adjusting the parameters of the directional distance of the roof-low roadway. A theory called “roof cutting of short wall beam” developed and improved leads to the technology of roof cutting and pressure relief that has substantially enhanced the efficiency of WF replacement^16–19^. Wu^20^ used a simulation to obtain the distortion law of surrounding rocks of gob-side entry retaining and proposed that roadside filling could make the deformation degree of the roadway smaller. Qin et al.^21^ used the FLAC3D software to research the stress distribution of mine pressure in gob-side entry retaining. That the stress field of the WF is asymmetric was concluded when the gob-side entry retaining had a mining mode, and the stress presented a U-shaped distribution in coal seams. Jiong^22^ conducted a simulation to examine the situation of air leakage when different “Y” type ventilation modes are in use and obtained the variation law of the “three zones” range of the goaf with the volume of air leakage. Gou^23^ resolved the accumulated gas issue in the upper corner of the “Y” type by establishing the mathematical equation to control the law of gas migration. Ganbo^24^ adopted a gas control scheme combining the features of long-distance, large-aperture, high-position fracture drainage, borehole drainage, and goaf blockage concerning the characteristics of the coal mine. Wei^25^ employed the FLUENT software to examine the variation of the spontaneous combustion region when the different parameters of nitrogen injection are under consideration. When compared with the conventional “Y” type, a roadway-based “Y” type ventilation using roof cutting and pressure relief consists of broken rocks or coal blocks. However, the formation process and the materials of the composition differ^26–29^. So, the law of gas transportation changes, causing the flow of fresh air to continuously move into the goaf of the WF. Thus, there exist significant differences in the flow field and the characteristics of air leakage of the goaf. Zhenbin^30^ utilized Fluent software to simulate the flow field of the goaf under the mining mode of roof cutting and pressure relief of gob-side entry retaining. The outcomes showed that the GC near the contact roadway was the highest, and the air leakage near the WF was found to be the area with severe air leakage. Shang^31^ explored the failure mechanism of overlying strata and the law of gas migration by conducting experiments and simulations. Fang^32^ studied the effect of N_2_ on CH_4_ desorption by a method called molecular dynamics. N_2_ pressure was positively correlated with methane desorption was concluded. Tang^33^ obtained the impact of gas extraction on air leakage in Goaf by running a simulation study. A third-order polynomial model represented the relation between gas extraction and air leakage. Besides, the air leakage also altered in a range. Through numerical analysis and other similar methods, Wenyue^34^ explored the problem of large deformation of gob-side entry retaining in large mining heights of WF and researched the effect of the coal pillar width on roadway deformation. Yudong^35^ provided an overview of the current situation and the development trend of the 110&N00 mining method and conducted detailed research applying the conditions of different mining methods at each stage. In addition, the goaf and the gob-side entry retaining were formed through a channel, and the goaf presented an open space. However, more in-depth research related to the law of air leakage in Goaf is needed and the control measures of gas overrun have been examined.

Therefore, the ventilation mode of the 1201 fully mechanized face in the south Wu mining area of the Daxing coal mine was adjusted by using the CFD numerical simulation when the technology of roof cutting, and pressure relief was premised. So, the ventilation mode of the fully mechanized mining face was altered from “U” to “Y” type, and the impact of the WF ventilation system and goaf flow field were studied. The ventilation scheme and the law of gas migration in the completely mechanized mining face in the Daxing coal mine were attained by applying roof cutting and pressure relief technology. Thus, the solution to the possible problems related to safety was demonstrated. The results would effectively assure the safe production of the mine and provide a reference for the treatment of gas overrun.

## Engineering background

The location of the Daxing coal mine is in the southwest of Tiefa Coalfield in Tieling City, Liaoning Province. When mining levels are under consideration, while the first mining level is located at −600 m, the second level is located at −800 m. The South Five 1201 WF is situated in the east of the South Five mining location at the first level. The WF has a minable strike whose length and width are 1221 and 195 m, respectively. The area of the WF is 238095 m^2^. The non-cohesive coal in the coal seams whose thickness is 1.42–2.35 m consists of 1–5 layers of rock. The general coal thickness is 1.84 m. The medium sandstone and mudstone whose thickness is 0.08–0.81 m comprise the rock’s lithology.

**Figure 1:**
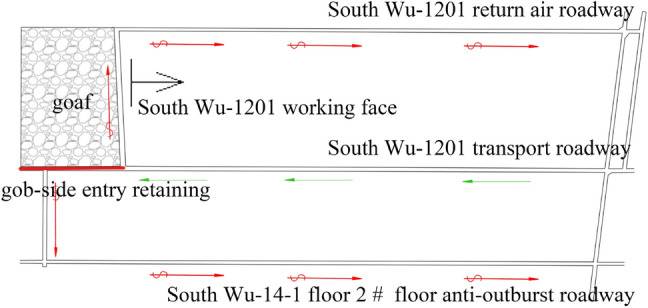
The Working Face’s Layout of The South Wu-1201. Figure 1 depicts the Working Face’s Layout, which is a “Y” type ventilation, of The South Wu-1201. The air is supplied by the transport roadway, and the 14–1 layer of the 2 # floor of the anti-outburst roadway and the return air roadway of the Nanwu-1201 return air.

To improve the replacement efficiency of the WF and increase the output, the utilization and adoption of roof cutting, and pressure relief technology are proposed to alter the 1201 completely mechanized mining face and make it into a ventilation mode type of “Y”. The ventilation of the WF was carried out by the 12th track middle roadway. The airflow was divided into two parts at the junction of the transport roadway and the WF. Most of them entered the WF and flowed into the return air roadway and the connection of the 12th return air roadway. The other small part of the gob-side entry retaining through the anti-outburst roadway was contacted with the roadway into the floor of the 14-1floor 2 # floor anti-outburst roadway. The airflow moving through the WF and the return air eye and the WF converged again. The ventilation network of the WF is a typically simple diagonal network, as shown in Fig. 1. The flow field has dynamic characteristics in the goaf that are affected by the changes in the process of advancing the WF, resulting in a frequent overrun of GC at the upper corner.

## The construction of the model

According to the node pressure energy of the goaf ventilation system and the geological data of the goaf in the Daxing coal mine, the spatial model was established, and the flow field in different production periods was simulated and analyzed. The influence law of air leakage and gas migration in the goaf on GC in-floor gas extraction roadway and the upper corner of WF was obtained.

### The establishment of the physical model

**Figure 2:**
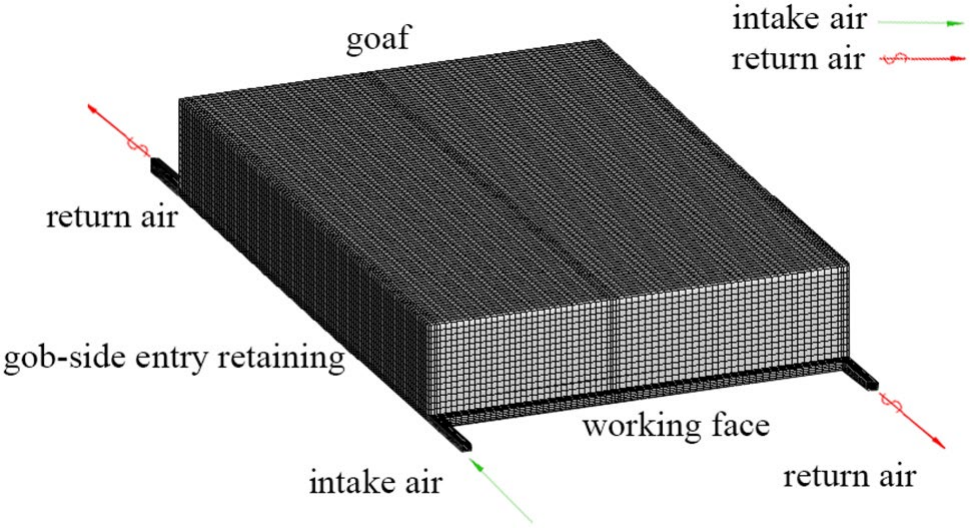
The Goaf Model of the South Wu-120. Figure 2 depicts the goaf model of the South Wu-1201. The length, width, and height are 280, 200, and 50 m, respectively. The shape of the inlet and return sections of the air roadway is a rectangle whose width and height are 4 and 4 m, respectively. The roadway is defined as a fluid domain. However, the air leakage inside the goaf is only considered a porous medium model.

The model called turbulence standard k-ε was selected and the goaf model having a 280-m length, 200-m width, and 50-m height was established. The shape of the inlet and return air roadway section is a rectangle whose width and height are 4 and 4 m, respectively. Figure 2 depicts that the WF, goaf, and roadway are defined as the fluid domain, and the air leakage inside the goaf is only considered. Thus, the assumptions below as a porous medium model are made:

1. Incompressible gas is assumed, and the flow state conforms to the seepage law.
2. The WF gas is single-phase isothermal.
3. The shape of the model space is simplified to a rectangle.
4. The gas flow in the WF conforms to Darcy's law.
5. The goaf is considered a heterogeneous porous medium, and the influence of gravity on the simulation results is ignored.

### The establishment of the mathematical equation

The air inlet is set as the velocity inlet. The volume of the whole air is 1300 m^3^/min. The air outlet is set as a free flow. The functional relationship between porosity and spatial position is defined by

1 $$n(x,y,z) = \left( {0.2e^{ - 0.0221x} + \, 0.1} \right)\left( {e^{{ - 0.15\left( {\frac{L}{2} \pm Z} \right)}} + 1} \right) \times 0.98$$

Equation (1) denotes the porosity at the point denoted by (x, y, z) in the goaf; L denotes the inclined length of the WF, m.

The seepage continuity is defined by

2 $$\frac{{\partial \left( {\rho \phi } \right)}}{{\partial {\text{t}}}} + \nabla \times \left( {\rho V} \right) = q\rho$$

where ρ represents the fluid density, kg/m^3^; φ denotes porosity, %; t is time, s; V represents the seepage velocity, m/s; q denotes the intensity of the source.

The gas pressure is a function of the coal seam changing based on time and space defined by

3 $$\frac{{\partial p^{2} }}{{\partial {\text{t}}}} = S\left( P \right)\left[ {\frac{{\partial^{2} P^{2} }}{{\partial r^{2} }} + \frac{1}{r}\frac{{\partial P^{2} }}{\partial r}} \right](t > 0,r_{0} < r < R)$$

4 $$S\left( p \right) = 2p_{n} p\lambda /\left[ {n + abcp\left( {2 + bp} \right)/\left( {1 + bp} \right)^{2} } \right]r^{2}$$

where λ denotes the coefficient of coal seal permeability, m^2^/(Pa2·s) ; r represents the radius variable of the radial flow field, m; pn denotes the standardized atmospheric pressure, Pa; t represents the time variable, s; a represents the highest score of gas absorbed by the coal body, m^3^/t; b represents the gas adsorption constant by the coal body, Pa-1; n denotes the free gas amount contained in the unit volume of coal, m^3^/m^3^; c represents the quality parameters of the coal.

The UDF programming for relevant parameters was carried out and loaded to Fluent software to resolve in combination with the measured data of the mine.

## Data analysis and simulation results

### The analysis of air leakage

When compared to the conventional “Y” type ventilation, the roof cutting, and pressure relief differ in air leakage due to the different filling processes and the composition of the roadway rock wall. The goaf and the retaining roadway are directly connected. The goaf is in an open space, and the flow field in the goaf is significantly different from the conventional “Y” type ventilation. The flow field in the goaf of them was studied and analyzed based on roadway deformation. The ventilation system of the WF in different production periods was constructed when the conditions of completely mechanized mining with the implementations of roof cutting, pressure relief, and roadway retention were in use. The change of the pressure energy of each important node when the “Y” type ventilation mode of roof cutting, pressure relief, and retaining roadway is in use, was analyzed based on the basic parameters of the roadway and the WF ventilation network theory. The change of the pressure energy of the goaf as the angular branch with time and space evolution of the WF was obtained, and the possible air leakage was analyzed.

**Figure 3:**
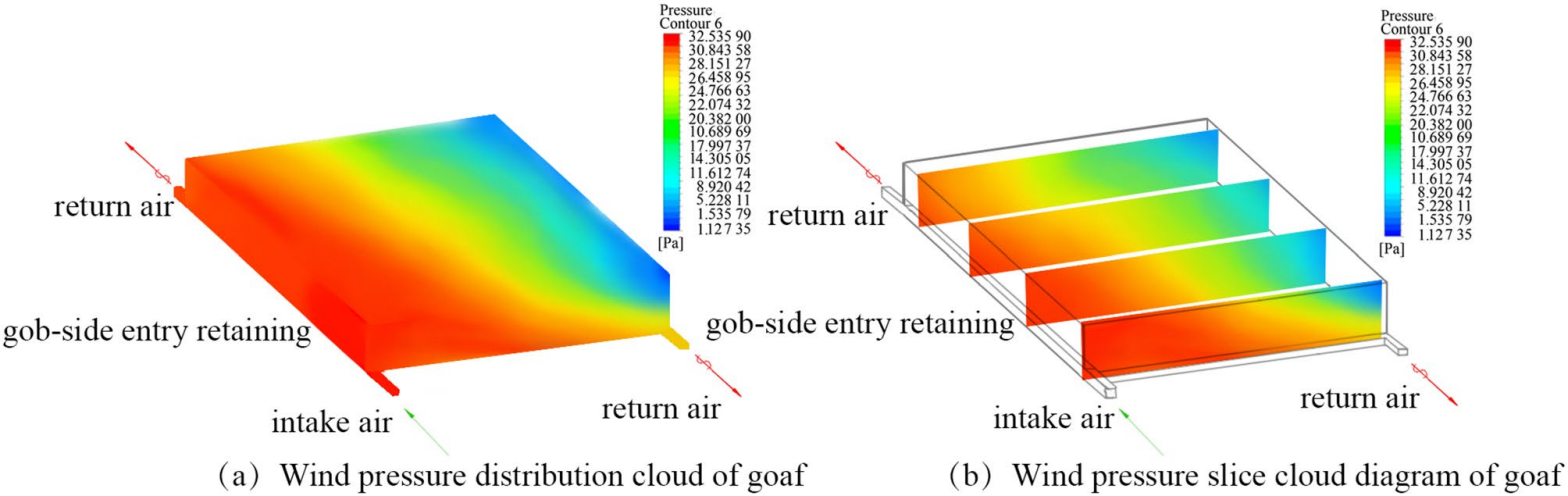
The Cloud diagram simulating the wind pressure of the goaf. Figure 3 depicts the cloud diagram of the wind pressure of the simulated by using the CFD. The pressure on the side of the roadway of the air inlet reaches a maximum of 32.53 Pa when the initial wind speed occurs. Then, the lowest point of pressure reaches 1.12 Pa at the upper corner of the working face. Because the retaining roadway is directly connected to the goaf, the pressure difference between the roadways of the air inlet and the air return roadway would become larger.

Figure 3 depicts the cloud map of the wind pressure of the goaf when the mode of roof cutting and pressure relief along the goaf is under consideration. While the volume of the air at the WF was supplied according to 1500 m^3^/min, the air supply at the floor anti-outburst roadway was 360 m^3^/min. The simulation parameters are called the strike length of the goaf, the porosity, the air volume of the return air roadway, and the air volume of the inlet air roadway are assigned to 280 m, 0.3, 11.52 m^3^/s, and 25 m^3^/s, respectively. Fresh air flows through the WF, moves into the return air roadway, and finally flows into the floor outburst prevention roadway. The pressure at the air inlet roadway has a maximum value when the initial wind speed measured is equal to 32.53 Pa. While the pressure is lower in the direction of the goaf near the return air roadway, the pressure in the direction of the WF gradually decreases on the route of the incoming wind. The lowest pressure point, 1.12 Pa, is measured at the upper corner of the WF. The utilization of roof cutting and pressure relief of the gob-side entry retaining makes the roadway surrounding rock pressure of the mining area raise, leading increase in air leakage. When the flow field in the goaf is under consideration, the pressure energy decreases from the retaining and the transport roadways to the goaf. Thus, the pressure difference between the inlet and the return air roadways could reach 31.41 Pa. Therefore, the air leakage mainly flows out through the cracks in the wall of the roadway since the retaining roadway and the goaf are directly connected. The goaf forms an open space, increasing the pressure of the retaining roadway.

By simulating the mine ventilation system of the 1201 WF of South Wu, the direction and amount of air leakage in the mining and extraction areas concerning different mining periods of the WF were obtained. Table 1 presents the parameters of the roadway. The coefficient of the frictional resistance, α, of the corresponding roadway could be attained by using the method called lookup table applied to the measured data of the mine.

**Figure 4:**
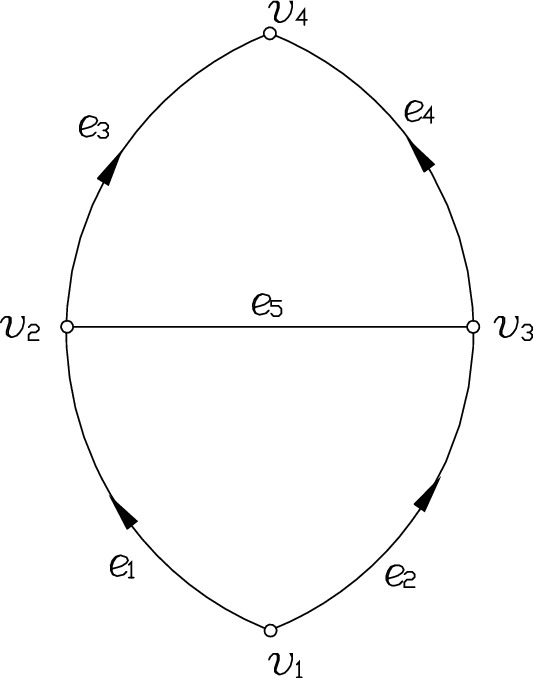
The Working Face’s Ventilation Network Diagram of the South Wu-1201. Figure 4 illustrates Working Face’s simplified ventilation network of the Nanwu 1201. V_1_ represents the junction point of the working face and the transportation roadway. V_2_ denotes the intersection point of the transportation roadway and the prevention roadway of the floor outburst. V_3_ represents the intersection point of the WF and the roadway of the return air. V_4_ represents the intersection point of the air return eye in the contact roadway of the 12th floor. Besides, the air leakage between the middle roadway track of the 12-layer and the roadway of the return contact is ignored.

Figure 4 depicts the simplified ventilation network of the Nanwu 1201 WF. V_1_ represents the junction point of the WF and the transportation roadway, V_2_ denotes the intersection point of the transportation roadway and the floor outburst prevention roadway, V_3_ represents the intersection point of the WF and the return air roadway, V_4_ represents the intersection point of the return air eye in the contact roadway of the 12th-floor return air. Besides, the air leakage between the 12-layer track middle roadway and the return contact roadway is ignored.

The ventilation network diagram of the 1201 WF of the Nanwu coal mine is described as follows:

5 $$\left\{ \begin{gathered} V = \left\{ {v_{1} ,v_{2} ,v_{3} ,v_{4} } \right\} \hfill \\ E = \left\{ {e_{1} ,e_{2} ,e_{3} ,e_{4} ,e_{5} } \right\} \hfill \\ R = \left\{ {r_{1} ,r_{2} ,r_{3} ,r_{4} ,r_{5} } \right\} \hfill \\ Q = \left\{ {q_{1} ,q_{2} ,q_{3} ,q_{4} ,q_{5} } \right\} \hfill \\ H = \left\{ {h_{1} ,h_{2} ,h_{3} ,h_{4} ,h_{5} } \right\} \hfill \\ \end{gathered} \right.$$

The loop direction is defined in a clockwise direction. According to the law of the loop resistance balance, Eq. (6)

6 $$\left\{ \begin{gathered} r_{1} q_{1}^{2} + r_{5} q_{5} \left| {q_{5} } \right| = r_{2} q_{2}^{2} \hfill \\ r_{3} q_{3}^{2} + r_{5} q_{5} \left| {q_{5} } \right| = r_{4} q_{4}^{2} \hfill \\ \end{gathered} \right.$$

In each airflow branch, e_1,_ e_2_, e_3_, e_4,_ and e_5_ represent the part of gob-side entry retaining, the WF, the three section roadways: floor outburst roadway, floor outburst prevention roadway, and return air eye, roadways: return air, return contact, and part of the 12th return air, and the air leakage channel of goaf connected by gob-side entry retaining and return air roadway, respectively. Since the retaining roadway intersects with the floor outburst prevention roadway at V_2_, the air leakage channel between the intersection point V_3_ of the WF and the return air roadway is the longest, and the wind resistance is also the largest. Therefore, the direction of e_5_ finds the overall flow direction between the gob-side entry retaining and goaf air leakage. Moreover, the direction of branch e_5_ relies on the relationship between the wind resistance $$\left\{ {r_{1} ,r_{2} ,r_{3} ,r_{4} } \right\}$$ and its adjacent branches $$\left\{ {e_{1} ,e_{2} ,e_{3} ,e_{4} } \right\}$$ and has nothing to do with the branch $$r_{5}$$. When $${\raise0.7ex\hbox{${r_{1} }$} \!\mathord{\left/ {\vphantom {{r_{1} } {r_{3} }}}\right.\kern-0pt} \!\lower0.7ex\hbox{${r_{3} }$}} = {\raise0.7ex\hbox{${r_{2} }$} \!\mathord{\left/ {\vphantom {{r_{2} } {r_{4} }}}\right.\kern-0pt} \!\lower0.7ex\hbox{${r_{4} }$}}$$, the flow volume of the branch $$e_{5}$$ is 0, when $${\raise0.7ex\hbox{${r_{1} }$} \!\mathord{\left/ {\vphantom {{r_{1} } {r_{2} }}}\right.\kern-0pt} \!\lower0.7ex\hbox{${r_{2} }$}} < {\raise0.7ex\hbox{${r_{3} }$} \!\mathord{\left/ {\vphantom {{r_{3} } {r_{4} }}}\right.\kern-0pt} \!\lower0.7ex\hbox{${r_{4} }$}}$$, The flow direction of the branch $$e_{5}$$ changes from V_2_ to V_3_, and when $${\raise0.7ex\hbox{${r_{1} }$} \!\mathord{\left/ {\vphantom {{r_{1} } {r_{2} }}}\right.\kern-0pt} \!\lower0.7ex\hbox{${r_{2} }$}} > {\raise0.7ex\hbox{${r_{3} }$} \!\mathord{\left/ {\vphantom {{r_{3} } {r_{4} }}}\right.\kern-0pt} \!\lower0.7ex\hbox{${r_{4} }$}}$$ the flow direction of the branch changes from V_3_ to V_2_.

**Figure 5:**
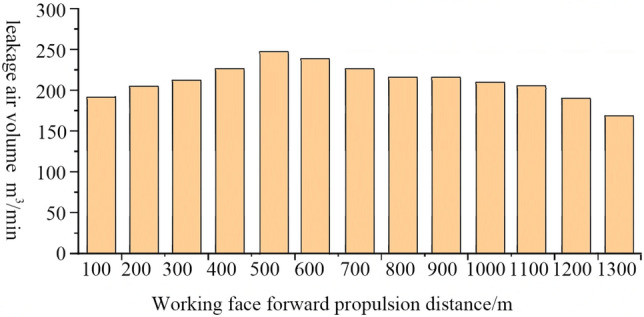
The volume of air leakage under different ventilation modes. Figure 5 illustrates the air leakage under retaining different lengths of gob-side entry by simulation. By adjusting the wind resistance of the lane of the roadway contact for the prevention of the floor outburst or the prevention roadway of the floor outburst. The airflow will move from the retaining gob-side entry to the goaf within the working face’s range of 0–500 m ahead. As the length of the retaining gob-side entry increases, the air leakage gradually increases. When the working face is pushed forward 500 m, the air leakage would be the largest amount of 247 m^3^ / min. When the range changes between 500 m through 1300 m, the air leakage would gradually decrease.

The simulation analysis gives the outcomes of air leakage when the different lengths of the retaining roadway are under consideration. Figure 5 depicts the results of adjusted wind resistance of the roadway contact lane for floor outburst prevention or floor outburst prevention roadway. Figure 4 depicts that the WF is advanced in the range of 0–500 m in the initial stage of the mining based on the adjustment of e3. When $${\raise0.7ex\hbox{${r_{1} }$} \!\mathord{\left/ {\vphantom {{r_{1} } {r_{2} }}}\right.\kern-0pt} \!\lower0.7ex\hbox{${r_{2} }$}}$$ less than $${\raise0.7ex\hbox{${r_{3} }$} \!\mathord{\left/ {\vphantom {{r_{3} } {r_{4} }}}\right.\kern-0pt} \!\lower0.7ex\hbox{${r_{4} }$}}$$, the pressure energy of node V_2_ is higher than that of node V_3_, and the airflow will move from the gob-side entry retaining to the goaf. When the length of the retaining roadway increases, the air leakage progressively raises. When the WF advances 500 m, the pressure energy of node V_2_ becomes higher than that of node V_3_ by 60.51 Pa. Then, the air leakage would be the largest, which is 247 m^3^/ min. The pressure energy difference between V_2_ and V_3_ nodes gradually decreases in the range of 500–1300 m and the air leakage decreases too. When the WF advances 1300 m, $${\raise0.7ex\hbox{${r_{1} }$} \!\mathord{\left/ {\vphantom {{r_{1} } {r_{2} }}}\right.\kern-0pt} \!\lower0.7ex\hbox{${r_{2} }$}}$$ becomes less than $${\raise0.7ex\hbox{${r_{3} }$} \!\mathord{\left/ {\vphantom {{r_{3} } {r_{4} }}}\right.\kern-0pt} \!\lower0.7ex\hbox{${r_{4} }$}}$$ , and the pressure energy of node V_2_ becomes higher than that of node V3, which is 48.95 pa. The airflow will move from the gob-side entry retaining to the goaf with an air leakage of 175 m^3^/ min. The roadway deforms, and the cross-sectional area, shape, and smoothness of the roadway will change due to the influence of mine pressure when the mining operation of the WF is in use. When the WF advances 1300 m and gets closer to the stop line, the roadway deforms to a certain extent. So, the friction coefficient is increased by 1.5 times, and the cross-sectional area is reduced to 85% of the original, which hinders the flow of air leakage.

### The analysis of gas concentration

**Figure 6:**
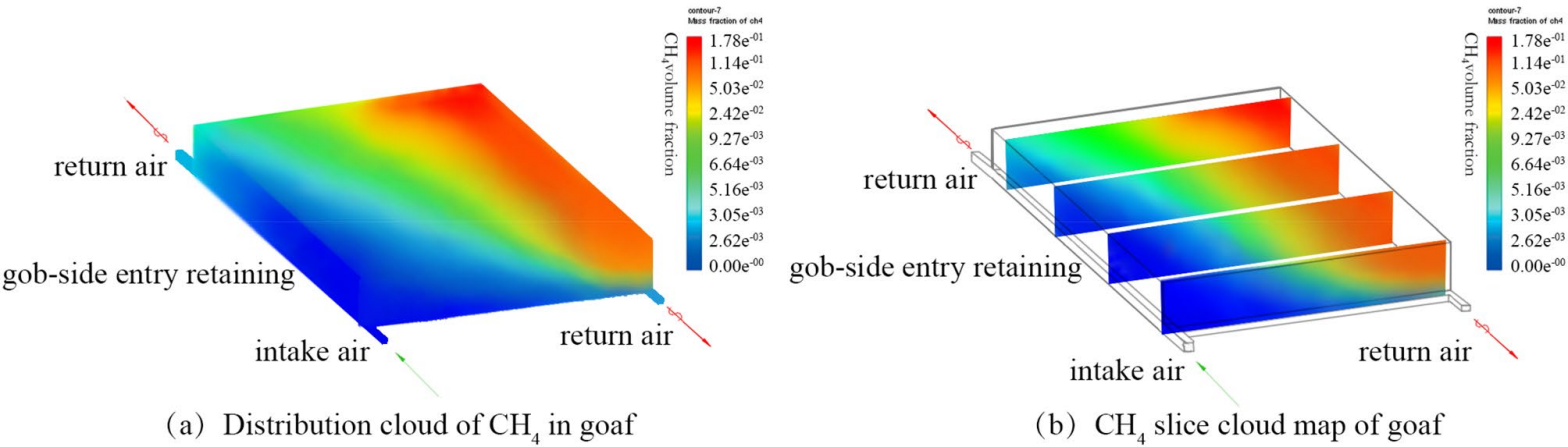
The Working Face’s simulation distribution of the gas concentration in the South Wu-1201 during mining. Figure 6 illustrates the simulation distribution of the gas concentration during the period of the mining. The gas concentration in the goaf gradually increases with the route of air intake. Due to the intensity of larger air leakage, the gas concentration would be small near the roadway of air intake. Under the retaining mining mode of gob-side entry with both roof cutting and pressure relief, the range of air leakage would be wider, and the intensity would be greater due to the open goaf space. Most of the air leakage flows from the retaining roadway to the goaf. The gas concentration in the deeper part of the goaf and the upper corner of the working face reaches 17.82% and 4.64% respectively. It is necessary to use gas control as the target.

The measured data show that the actual air leakage was found to be 238.67 m^3^/min, which is almost the same as the results of the simulation. The cloud map of the gas distribution is shown in Fig. 6, which opposes the oxygen concentration distribution. The GC in the goaf gradually increases with the air intake route. The GC equals almost 0 near the roadway of the air intake due to the air linkage’s large intensity. Since high-degree collapses occur and compactions are observed in the deep goaf, the gas leakage would be small, which leads to a high concentration of gas, reaching up to 17.82%. The goaf presents an open space when the mining mode of cutting, and pressure relief gob-side entry retaining is under consideration. Thus, most of the air leakage flows from the retaining roadway to the goaf, which alleviates the problem of gas accumulation near the retaining roadway. The retaining and transportation roadways had low concentrations, and the range of gas accumulation in the deep goaf was large. It mainly occurred in the deep goaf and the upper corner of the WF near the side of the return air roadway. Because the outlet is near the upper corner of the return air roadway, the gas is difficult to be discharged. So, accumulation occurred. Thus, high concentration was observed in a local area. The GC at the upper corner of the WF equals 4.64%, which far exceeds the safety standard of the coal production in the mine.

### The analysis of gas control

**Figure 7:**
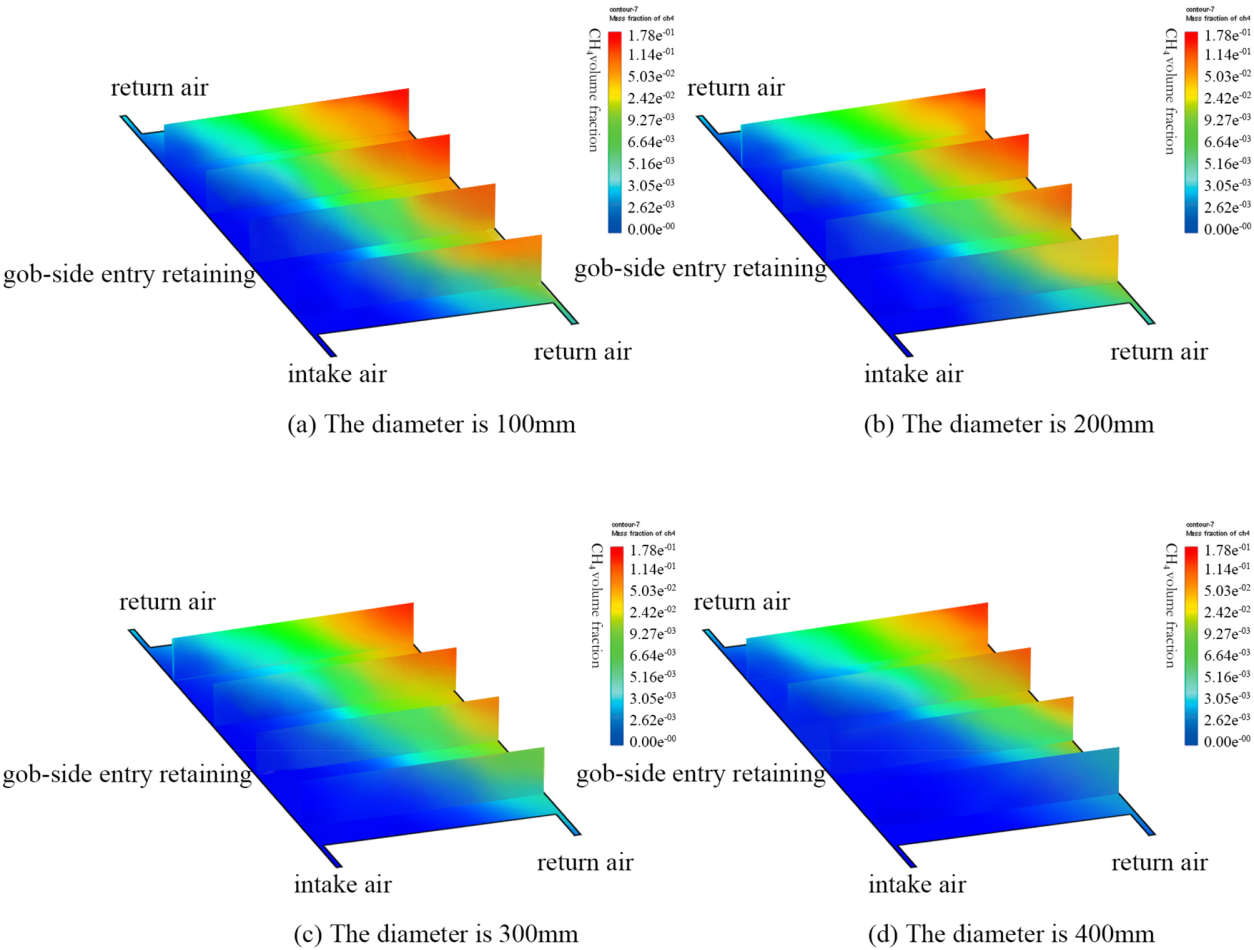
The cloud map of the gas concentration distribution of goaf when different diameters of the pipe are used. Figure 7 illustrates the cloud map of the gas concentration distribution under the different diameters of the pipe. When the diameter of the buried pipe reaches 100 mm, the gas concentration in the upper corner does not change significantly, which is still maintained at about 4.01%. When the diameter of the buried pipe reaches 400 mm, the gas concentration decreases obviously. The amount of gas flowing into the upper corner is effectively controlled, reaching 0.37%. At this time, the gas concentration in the deeper goaf would be still high, reaching 12.26%. Further measures need to be taken.

To resolve the issue of gas overrun in the upper corner, the manuscript concentrates on the investigations of the control effect of different buried pipe sizes on gas accumulation when the roof cutting and pressure relief gob-side entry retaining is modulated. Based on the actual production capacity of the mine, combined with economic benefits and construction difficulty, the treatment effect of simulated borehole sizes of 100 mm, 200 mm, 300 mm, and 400 mm were determined by calculating relevant parameters with the burial depth set at 4.0 m. Figure 7 depicts that the GC based on different sizes of pipe diameter shows a downward trend, and the effect of gas extraction in the upper corner is found to be positively correlated with the sizes of pipe diameter. The GC in the deep section of the goaf decreases slightly but the range of the change is not large. When the diameter of the buried pipe is set to 100 mm, the GC in the upper corner does not change substantially, which is still maintained at about 4.01%. Gas mainly flows into the upper corner location with air leakage, which fails to meet the specifications of safe production. On the other hand, when the diameter of the buried pipe was assigned to 200 mm, the gas in the upper corner decreased to 2.12% due to the influence of gas extraction. However, the GC in the deep section of the goaf does not change much. Moreover, when the diameter of the buried pipe is assigned to 300 mm, the GC in the upper corner is maintained at about 0.84%. Therefore, when the diameter of the buried pipe is set to 400 mm, the GC decreases greatly. The amount of gas flowing into the upper corner is effectively controlled at 0.37%. The gas in the deep section of the goaf can also be effectively extracted, but the GC is still high, which is 12.26%, and further measures are needed.

**Figure 8:**
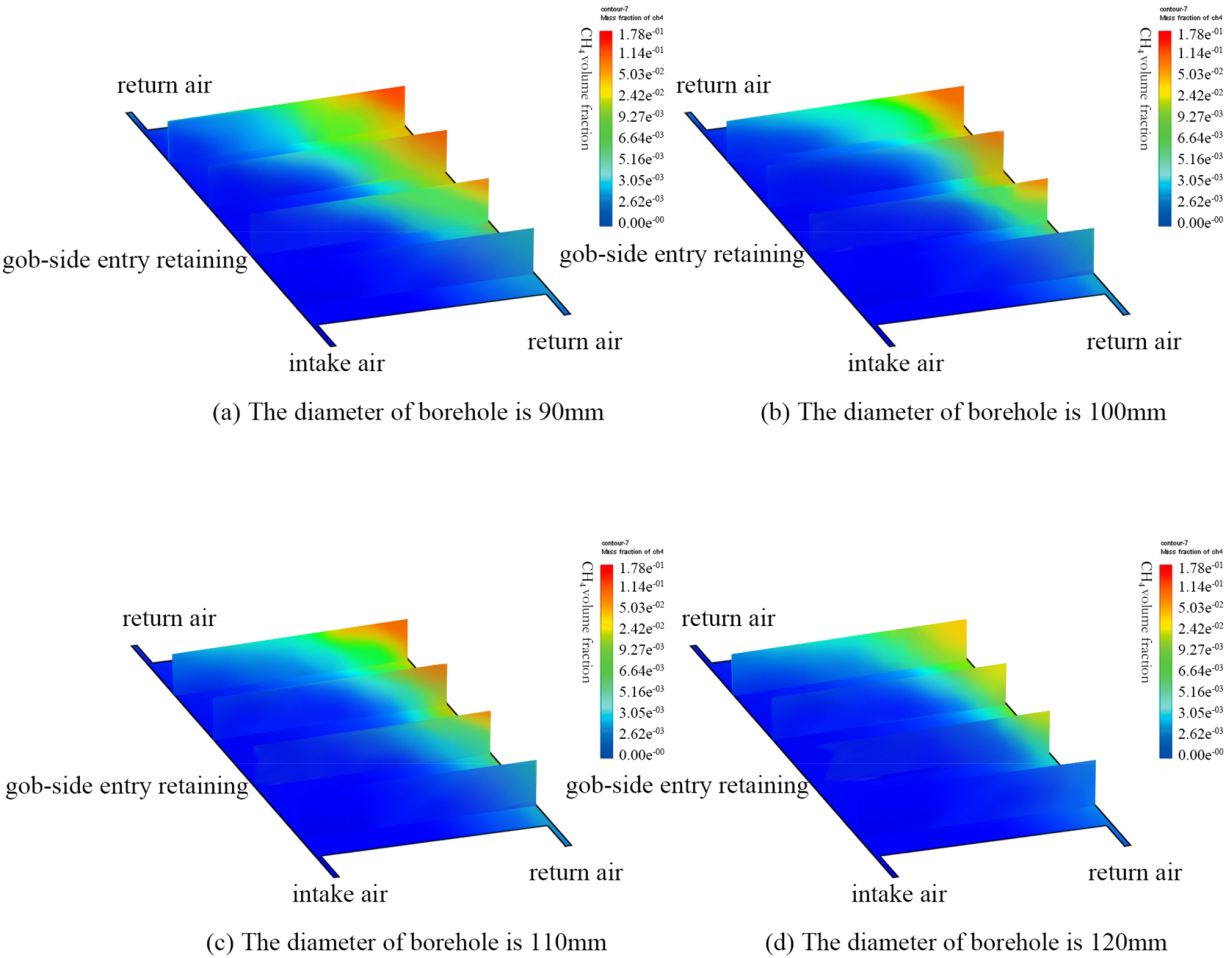
The cloud map distribution of goaf gas concentration is presented when the different diameters of the borehole are used. Figure 8 illustrates the cloud map of the gas concentration distribution in the deeper goaf under the different diameters of the borehole. To further resolve the problem of the higher gas concentration in the deeper goaf, the gas concentration in the goaf is simulated when the diameter and the depth of the buried pipe are 400 mm and 4.0 m, respectively, and the high-level borehole is carried out in the deeper goaf. When the diameter of the borehole is 120 mm, the gas concentration decreases most obviously. The maximum gas concentration in the deeper goaf decreases to 3.52%. The gas concentration in the upper corner is further decreased to 0.21%, ensuring the working face’s safe production.

To further resolve the issue of high GC in the deep goaf, the GC in the goaf is simulated by carrying out the high drilling in the deep goaf when the diameter and the width of the buried pipe are 400 mm and 4.0 m, respectively. Figure 8 depicts the treatment impact of the upper gas in the goaf when different drilling diameters are in use. The GC in the deep goaf decreases more obviously when the drilling diameter increases. The GC in the goaf changes little, which is 11.52%, and the extraction effect is not fine when the drilling diameter is assigned to 90 mm. On the other hand, when the diameter is assigned to 100 mm, the GC in the goaf begins to decrease. When compared with the prior reduction of 2.03%, the GC in the goaf is still high, and the alteration of GC at the upper corner of the WF is not obvious. When the diameter is set to 110 mm, the GC in the deep section of the goaf is 7.61%, and the GC in the upper corner is reduced to 0.34%. Because most of the gas in the deep goaf is extracted, only a small fraction of gas enters the WF due to air leakage, so the GC in the upper corner also decreases. When the diameter of the borehole is set to 120 mm, the GC decreases greatly. Thus, the maximum GC in the deep goaf lowers to 3.52%. Moreover, the GC in the upper corner is further reduced to 0.21%. The GC in the goaf is efficiently reduced and the safe production of the WF is ensured.

## Applications

**Figure 9:**
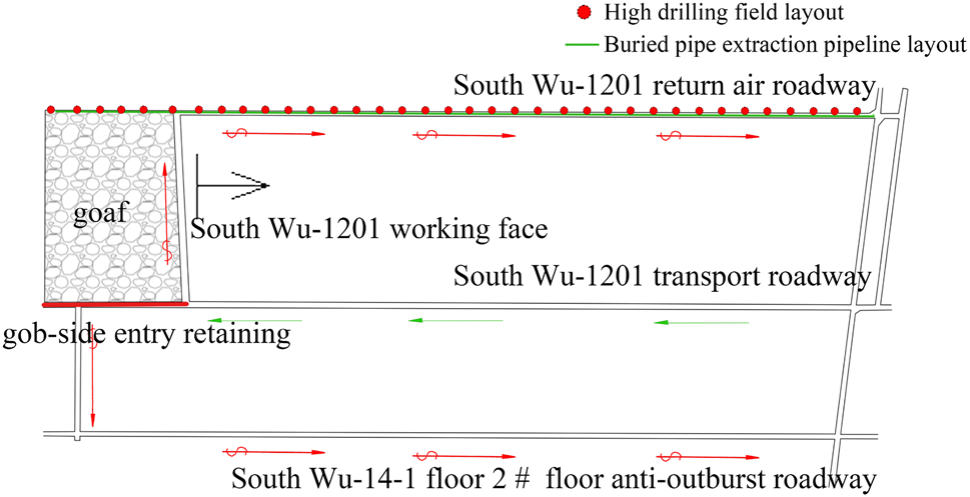
The extraction pipeline’s layout diagram. Figure 9 illustrates the actual layout of the extraction pipeline. Based on the above simulation and the actual production of the mine, To resolve the issue of gas overrun, Gas extraction is implemented to control the gas amount by conducting technical research in the Daxing coal mine. While the high-concentration gas system is used to extract high-level borehole gas, a low-concentration gas system is used to extract the upper corner gas of the WF.

Both the simulation study and the real production status of mine suggest that if the measures of gas control are not carried out, the gas overrun in the upper corner will trouble the production of the Nanwu 1201 WF. Especially, if the advancement of production is aimed, the gas emission in the goaf would increase, and lead to a prominent problem. To resolve the gas overrun issue, an implementation of gas extraction to control gas was decided by conducting technical research in the Daxing coal mine. While the high-level borehole gas is extracted by a high-concentration gas system, the upper corner gas of the WF is extracted by a low-concentration gas system. Figure 9 depicts the layout of the extraction pipeline.

A system of low negative pressure and high concentration extraction:

The system consists of high-level gas extraction boreholes and pipelines that are used to extract the upper high-concentration gas inside the goaf. When economic benefits and construction difficulties are combined, 6 boreholes are arranged in each drilling field whose spacing is 30 m. The spacing is 3 m. While the diameter of the borehole is 114 mm, its depth is 26 m. The pipeline is arranged in the south five 1201 return air roadway as follows: A high concentration extraction pipeline → Return air roadway ((DN300 mm pipeline) → the 12th of concentrated return air roadway in the east wing of the south five (DN400 mm pipeline) → the 12th of concentrated return air downhill in the south five (DN400 mm pipeline) → the 9th of track uphill in the south five (DN400 mm pipeline) → the 9th of return air middle roadway in the south five (DN400 pipeline) → 516 return air auxiliary roadway in the south five (DN400 pipeline) → 516 return air main roadway in the south five (DN400) → south well pumping station.

A system of high negative pressure and low concentration extraction:

Two low-concentration extraction pipelines are arranged in the South five 1201. The depth of the buried pipe is 4.0 m. The diameter of the pipe is 400 mm. So, Pipeline layout for Return air roadway → South five east wing 12th concentrated return air roadway → South five 12th concentrated return air downhill → South five 9th track uphill → South five south wing air distribution roadway → South air shaft → South well pumping station.

**Figure 10:**
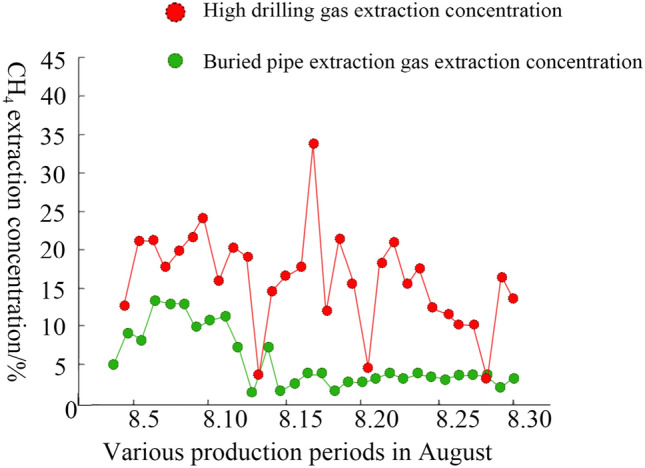
Line chart representing the concentration of gas extraction in August 2022. Figure 10 illustrates the extraction of the gas concentration in the line chart in August 2022. While the system extracting high-concentration gas reaches between 10 and 20%, the system extracting low-concentration gas reaches basically between 2 and 5%.

**Figure 11:**
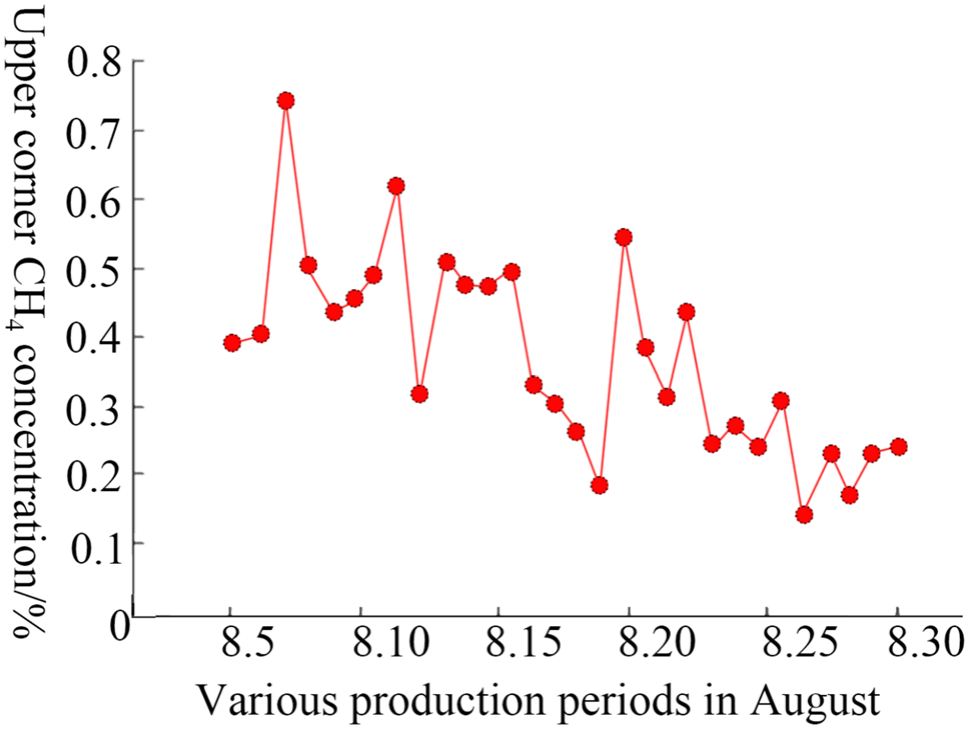
The distribution of gas extraction of the upper corner in August 2022 in the line chart Fig. 11 illustrates the distribution of gas extraction of the upper corner in August 2022. The gas concentration in the upper corner has always been below 0.8% but reaches the highest score of 0.74%. The gas problem can be resolved is shown after pre-extraction of high-level borehole gas. The gas extraction of buried pipes is realized in the Goaf before mining.

After the project is implemented, the diagram represented by broken lines depicting gas extraction concentration in August is shown in Fig. 10. After adopting the measures of using a high-concentration gas extraction system to extract high-level borehole gas and a low-concentration gas extraction system to extract the upper corner gas. The GC of the high-concentration gas extraction system changes between 10 and 20% except for the abnormal data observed on some days such as the 15th, 19th, and 28th of August corresponding to 38.64%, 4.31%, and 3.15%. On the other hand, the concentration of other points follows the regular interval. Moreover, it shows that the high-concentration extraction system works stably and maintains a stable amount, which has a good impact on controlling the accumulation of gas in the WF. The change in GC in a low-concentration gas extraction system is larger than that in a high-concentration gas extraction system. Before August 11, the concentration of gas extraction was above 4%. After August 14, the GC was between 2 and 5%. Before August 13, the concentration of GC was higher than the subsequent GC, which indicates that the gas emission in Goaf was larger before August 13. The gas in the upper corner and the deeper part of the goaf is effectively controlled is shown, which follows the result of the borehole whose diameter is 120 mm is used. The change of high concentration gas extraction tended to be higher than that in the first half of the month. Figure 11 depicts the monitoring curve of the GC in the upper corner whose score is lower than 0.8%. However, the highest measurement reaches 0.74% on the 6^th^ of August 6, which gives similar outcomes when compared to the results of the simulation. Thus, the issue of gas overrun is effectively resolved in the upper corner of the WF. It showed that gas migration under open goaf is not conducive to gas control, but the gas problem can be resolved basically by conducting pre-extraction efforts of high-level borehole gas and buried pipe gas extractions in goaf before mining when the condition of gob-side entry retaining by 110 methods is under consideration.

## Conclusion

The gas overrun issue in the goaf and upper corner was caused by serious air leakage in the goaf when the mode of “Y” type ventilation of gob-side entry retaining with roof cutting and pressure relief in the Daxing coal mine was in use. Thus, the air leakage law and gas control were researched. The characteristics of air leakage of “Y” type ventilation of gob-side entry retaining with roof cutting and pressure relief were obtained by conducting the CFD simulation. Finally, reasonable measures of gas extraction were formulated based on the problem of gas overrun caused by employing the scheme. The following conclusions are obtained:

1. The law of air leakage characteristics is obtained through simulation. The utilization of gob-side entry retaining with roof cutting and pressure relief technology makes the goaf structure an open space, which leads to the decrease of pressure in the retaining roadway. While the lateral wind pressure of the air roadway’s intake reaches the highest 32.53 Pa, the pressure of the upper corner of the WF reaches the lowest 1.12 Pa. By adjusting the wind resistance of the floor outburst prevention roadway contact lane or floor outburst prevention roadway, the pressure difference of airflow affects the airflow from gob-side entry retaining to goaf. So, the air leakage increases as the length of the retaining roadway raises. Therefore, the goaf and the retaining roadway form a complete airflow channel. When the WF advanced 500 m, the maximum air leakage was found to be 247 m^3^/min. When the WF was pushed further to 1300 m, the roadway was deformed due to the influence of mine pressure, the coefficient of friction increased by 1.5 times which hindered the air leakage. Thus, the minimum amount of air leakage was 175 m^3^/min.
2. For the gas overrun issue in the upper corner caused by air leakage, the extraction impact of the gas-buried pipe was positively correlated with the diameter of the buried pipe and was concluded by simulating the treatment effect of different extraction measures. When the depth and the diameter of the buried pipe are set to 4.0 m and 400 mm, respectively, the extraction effect is found to be the best. Besides, the GC in the upper corner is found to be 0.37%. However, the GC in the deep section of the goaf was found to be bigger, which is 12.26%. Besides, high drilling gas extraction is further implemented on this basis. When the diameter of the borehole is set to 120 mm, the GC in the goaf lowers greatly, which decreased to 3.52%, and the GC in the upper corner is found to be 0.21%, which met the relevant production norms of safety mining.
3. An investigation was carried out based on the measurement of the field data and numerical simulation. When combined with the real economic benefits, the Daxing coal mine decided to adopt two sets of gas extraction systems. While the high-level borehole gas is extracted by employing the high-concentration gas extraction system, the system extracting low-concentration gas is utilized to extract upper-corner gas. While the GC of the system that extracts high-concentration gas changes between 10 and 20% in the data detection process in August, the GC of the system that extracts low-concentration gas alters between 2 and 5%. The GC in the upper corner has always been less than 0.8%, which guaranteed the safe production of the mine. The issue of gas overrun in the upper corner was resolved and an important reference to control mine gas was provided.

## Data Availability

All data generated or analyzed during this study are included in this article.
